# Bioactive Compounds Obtained from Polish “Marynka” Hop Variety Using Efficient Two-Step Supercritical Fluid Extraction and Comparison of Their Antibacterial, Cytotoxic, and Anti-Proliferative Activities In Vitro

**DOI:** 10.3390/molecules26082366

**Published:** 2021-04-19

**Authors:** Katarzyna Klimek, Katarzyna Tyśkiewicz, Malgorzata Miazga-Karska, Agnieszka Dębczak, Edward Rój, Grazyna Ginalska

**Affiliations:** 1 Chair and Department of Biochemistry and Biotechnology, Medical University of Lublin, Chodzki 1 Street, 20-093 Lublin, Poland; malgorzata.miazga-karska@umlub.pl (M.M.-K.); g.ginalska@umlub.pl (G.G.); 2 Supercritical Extraction Department, Łukasiewicz Research Network- New Chemical Syntheses Institute, Al. Tysiąclecia Państwa Polskiego 13a, 24-110 Puławy, Poland; agnieszka.debczak@ins.lukasiewicz.gov.pl (A.D.); edward.roj@ins.lukasiewicz.gov.pl (E.R.)

**Keywords:** hop, *Humulus lupulus* L., supercritical extraction, Marynka strain, xanthohumol, antibacterial activity, cytotoxicity, anti-proliferative activity

## Abstract

Given the health-beneficial properties of compounds from hop, there is still a growing trend towards developing successful extraction methods with the highest yield and also receiving the products with high added value. The aim of this study was to develop efficient extraction method for isolation of bioactive compounds from the Polish “Marynka” hop variety. The modified two-step supercritical fluid extraction allowed to obtain two hop samples, namely crude extract (E1), composed of α-acids, β-acids, and terpene derivatives, as well as pure xanthohumol with higher yield than that of other available methods. The post-extraction residues (R1) were re-extracted in order to obtain extract E2 enriched in xanthohumol. Then, both samples were subjected to investigation of their antibacterial (anti-acne, anti-caries), cytotoxic, and anti-proliferative activities in vitro. It was demonstrated that extract (E1) possessed more beneficial biological properties than xanthohumol. It exhibited not only better antibacterial activity against Gram-positive bacteria strains (MIC, MBC) but also possessed a higher synergistic effect with commercial antibiotics when compared to xanthohumol. Moreover, cell culture experiments revealed that crude extract neither inhibited viability nor divisions of normal skin fibroblasts as strongly as xanthohumol. In turn, calculated selectivity indexes showed that the crude extract had from slightly to significantly better selective anti-proliferative activity towards cancer cells in comparison with xanthohumol.

## 1. Introduction

Hop (*Humulus lupulus* L.), due to its rich composition, has long been a valuable raw material used in various industries and still arouses the interest of research centers. Hop cones are the source of valuable bioactive compounds from the group of polyphenols, essential oils, monosaccharides, amino acids, proteins, lipids, fatty acids, pectins, salts, lignins, water, and bitter acids (commonly used in brewing) [1,2,3].

Among hop polyphenols, the most important group are prenylflavonoids, including xanthohumol (XN), its isomer isoxanthohumol (IXN), and desmethylxanthohumol (DMXN) as well as its derivatives: 8-prenylnaringenin (8-PN) and 6-prenylnaringenin (6-PN) [3,4,5]. It is worth noting that xanthohumol is most often used in the chemistry, pharmacy, and medicine. It constitutes 80–90% of all prenylflavonoids found in hops and its concentration varies, depending on the variety, from 0.2% to 1.3% of dry matter [2]. Xanthohumol is characterized by a unique biological activity. It primarily exhibits anti-inflammatory and antioxidant properties [6,7,8]. This compound also shows anti-cancer activity at all stages of carcinogenesis (initiation, promotion, and progression) for cancers of breast, intestine, ovary, prostate, multiple myeloma, and lymphocytic leukemia [8,9,10,11]. Xanthohumol also has antifungal, antibacterial, insect antifeedant, and antiviral properties [5,7,12,13,14,15].

Besides polyphenols, bitter acids constitute very interesting group of hop components. The most important bitter acids are the acids α- (humulones) and β- (lupulones), which are prenyl derivatives of floroglucin. They can occur in the form of oils or resins easily soluble in organic solvents. Depending on the acyl side chain, five analogs are distinguished, namely n-, co-, ad-, pre-, post-humulone/lupulone [1,16]. The main components of α-acids are analogs of n-humulone (35–70% α-acids), co-humulone (20–65% α-acids), and ad-humulone (10–15% α-acids). Among the analogs of β-acids, there are lupulones (20–55% of β-acids), co-lupulones (20–55% of β-acids), and ad-lupulones (10–15% of β-acids), respectively [3,16]. Furthermore, hop cones contain small amounts of posthumulone, prehumulone as well as adprehumulone. Humulone exhibits sedative/hypnotic effects and acts as a positive allosteric modulator of GABA_A_ receptors. This supports humulone’s substantial role in hops sleep-promoting activity and brings further insight into the probable mode of action for this behavior [17]. The α-acids show mild antiseptic activity against Gram-positive bacteria, including *Lactobacillus* sp., *Streptococcus* sp., *Staphyllococcus* sp., or *Bacillus* sp. On the other hand, hop β-bitter acids exhibit antidepressant-like effects in vitro. They also possessed antimicrobial activity [1,14,16,18,19].

It is also worth mentioning the essential oils in hop. The most desirable compounds that give a pleasant hop aroma include humulene and its oxidation products, such as humulene oxides. Humulene and myrcene are the most concentrated, therefore the ratio of both in the essential oil is an important quality feature characteristic of the variety. Humulene, is a naturally occurring monocyclic sesquiterpene (C_15_H_24_) [20,21]. The α-humulene is a sesquiterpene with known anti-inflammatory activity [22,23]. The β-caryophyllene, is a natural bicyclic sesquiterpene that is a constituent of many essential oils of hops, hemp (*Cannabis sativa*), rosemary. The European Food and Safety Authority (EFSA) also considers it safe and allows it to be used in the form of a food additive, flavor enhancer, cosmetic additive or flavor additive. It can be used to treat pain and inflammation [24,25]. Myrcene, β-farnesene, and β-cariophylene can be used to combat bee disease e.g., *V. destructor* mite [26]. All the above-mentioned chemical compounds are characterized by a strong antimicrobial effect [27,28,29].

Taking into account health-beneficial properties of compounds from hop, there is a growing trend towards developing successful extraction methods with the highest yield, but also receiving the products with high added value. In this case, the supercritical fluid extraction (SFE) method is considered as the most effective one in the extraction of a number of bioactive compounds [1,2]. The literature data indicate that SFE is widely used to extract various raw materials, including food-by-products, seaweeds, microalgae [30], herbaceous plants [31], fruits, and vegetables [32]. What is more interesting, the hop extraction, previously done with ethanol, has been almost entirely dominated by supercritical technology. Due to the non-polar nature of carbon dioxide, it is mainly used for the extraction of lipophilic compounds [32]. However, it is also possible to obtain resins and polyphenols by the extraction with carbon dioxide in a supercritical state. Since polyphenols are polar compounds, a small amount of polar co-solvent is necessary to be added to the CO_2_ stream in order to increase its polarity. Importantly, the key issue in performing the extraction is the appropriate selection of the extractant. The solvents should be chosen according to their selectivity towards one chemical compound or group of compounds and not (or to a negligible extent) to the others. The effectiveness of the liquid extraction process depends mostly on the temperature and the intensity of mixing the raw material and the extractant [1,21]. Thus, the choice of appropriate parameters is crucial during the process and strictly influences the extraction yield.

The aim of this work was to develop and characterize a modified two-step supercritical fluid extraction method for obtaining compounds from Polish “Marynka” hop variety. The purpose of this study was also to evaluate biological activities of obtained compounds. Resultant samples of crude extract (E1) and pure xanthohumol were subjected to an evaluation of antibacterial (namely anti-acne as well as anti-caries), cytotoxic, and anti-proliferative properties. Finally, their therapeutic safety in vitro and selective anti-proliferative activity towards normal and cancer cells in vitro were determined.

## 2. Results and Discussion

### 2.1. The Hop Cones Extraction

According to Hrnčič et al. [33], a number of studies have been performed in the past two decades towards the separation of bioactive compounds from hop cones as well as the production of hop extracts-based formulations. The commonly used solvents for the extraction involve ethanol, methanol, ethers, acetone, and others [34]. However, the conventional extraction methods, such as PLE (pressurized liquid extraction) or Soxhlet, require additional step for the removal of solvents but also the analysis of extracts purity for the presence of solvent residues [35]. Carbon dioxide is a commonly used solvent for the extraction and separation of plant extracts, especially resin and oil fractions. The first patents and works describing the use of CO_2_ in a liquid or supercritical form date back to the late 1970s [36,37]. The extraction of CO_2_ in a supercritical state has some advantages over other methods, as it enables the flexible change of process parameters [38], increases the dissolving power [39], increases the extraction rate [40], and allows for the production of extracts with a higher content of volatile and bitter substances [41].

To date, the two-step supercritical fluid extraction for Magnum, Hallertau Tradition, Spalt Selekt, Aroma, and K-62 hop cones has been performed by Zeković et al. [42]. The first step involved the use of 40 °C and 150 bar for 2.5 h, whereas the second step was studied at the temperature of 40 °C and the pressure of 300 bar. The highest extraction yield was obtained for Magnum, amounting 13.35 g and 7.54 g per 100 g of raw material using 150 and 300 bar, respectively. The extraction yield for other cultivars was in the range of 6.18–9.09 g per 100 g of raw material in a first step (150 bar) and 2.31–3.59 g per 100 g of raw material in a second step (300 bar) [42].

In our study, two-step extraction process was applied for the extraction of Marynka hop cones in terms of extraction of α-acids, β-acids, and essential oils compounds in a first step (CO_2_, 50 °C, 300 bar) and xanthohumol in a second step (CO_2_/EtOH, 85 °C, 800 bar). The applied extraction method allowed to obtain crude extract (E1) (see Figure 2) with extraction yield equal to 11.40% by weight. Another literature report indicated that 50 °C, 250 bar, solvent/raw material ratio of 1:1 and 80% of ethanol was optimal conditions for the extraction of flavonoids from granulated hop cones [43]. It was observed that the higher the ethanol concentration, the greater the extraction yield. However, based on a relatively high concentration of ethanol as a modifier of carbon dioxide, it may be assumed that the extraction of flavonoids was performed under subcritical conditions [44]. In comparison with a previous study performed by Rój et al. [1], the evaluation of influence of solvent (pure carbon dioxide or carbon dioxide modified with ethanol (1.0 wt%)) on the content of xanthohumol was performed. As it occurred, the influence of pure carbon dioxide on the content of xanthohumol was higher than that with modified carbon dioxide (6.50 vs. 4.80 wt%). However, taking into consideration the composition of spent hops extracts in terms of phenolic compounds, the higher total phenolic content (TPC) was analyzed in the extract obtained with carbon dioxide and ethanol than with pure carbon dioxide, as described in the next section.

### 2.2. The Chemical Composition of Hop Cones Fractions

In this study, the predominant components of crude extract (E1) were myrcene—(20.51%), β-caryophyllene—(4.88%), β-farnesene—(4.69%), and humulene—(18.3%) with the highest share (Table 1). According to literature, hop cones scCO_2_ (supercritical carbon dioxide) extracts were mainly composed of terpene derivatives including monoterpene hydrocarbons (7.4%), oxygenated monoterpenes (1.9%), sesquiterpenes hydrocarbons (24.7%), oxygenated sesquiterpenes (9.5%), diterpene, triterpene, steroids (10.7%), and phloroglucinol derivatives (24.8%) [1].

In their studies, Zeković et al. [42] obtained α-acids in hop cones extracts in the range of 9.8–41.0 wt%. The highest amount of α-acids was detected in Magnum extract. However, the highest content of α-acids was obtained when two-step extraction was applied. In the presented study, the one-step extraction resulted in the same amount of α-acids (41.0 wt%) as obtained by Zeković et al. [42]. The content of α-acids and β-acids was approx. 42% *m/m* and 19% *m/m*, respectively. The results of chemical composition analysis are presented in Table 2.

The supercritical hop extract contains almost all essential oils contained in hops, as well as a high ratio of α- to β-acids, which enables the production of beer with a good balance of hop aroma and bitter taste [43,45]. An important by-product of supercritical extraction is spent hops. This biomass residue is widely used as a fertilizer due to its high nitrogen content. To increase added value, the sustainable use of spent hops is an important goal in the hop processing industry. Under supercritical extraction conditions used to prepare extracts for breweries (pressure: < 300 bar, temperature: 40–60 °C), polar compounds, such as flavonoids, remain in the waste material [43,46]. The most abundant and widely studied component identified in used hops is prenylated chalcone, i.e., xanthohumol (XN) [1,19,45]. To produce an extract enriched with xanthohumol, post-extraction residues R1 (see Figure 2) were used after the extraction of bitter acids and essential oils, which contained approx. 0.4% XN in a relation to dry weight. The residues R1 were re-extracted at the pressure of 800 bar and the temperature of 85 °C. As a result of re-extraction, an extract enriched with XN to the level of approx. 4.80% was obtained (Table 2). In the recent studies, Grudniewska and Popłoński [47] applied chlorine-based DESs (deep eutectic solvents) as a simple and green method for the extraction of xanthohumol from spent hops. The highest yield was demonstrated for choline chloride and propylene glycol in the ratio of 1:2 (mol/mol), but it was over 20-times lower in comparison with the result obtained in this study (0.23 wt% vs. 4.80 wt%). Another literature data [45] reported 4-times lower xanthohumol content (1.23%) under similar conditions (80 °C and 850 bar) with pure CO_2_. In fact, the lower xanthohumol content may not be in accordance with the studies performed by Kostrzewa et al. [46] that the solubility of xanthohumol increases with the increase of the extraction pressure. The extract E2 obtained with the mixture of CO_2_/EtOH (99:1, *v/w*) was used further for xanthohumol purification with CPC. According to UV-Vis spectrometer analysis of TPC, extract E2 was characterized by higher content of phenolic compounds in total (52.19 mgGAE/g extract) than extract E1 (48.71 mgGAE/g extract). As it was observed, the solvent (CO_2_/EtOH) chosen for 2-step extraction had a positive influence on the content of these bioactive compounds.

The potential of liquid-liquid preparative chromatography including CPC (centrifugal partition chromatography) and CCC (countercurrent chromatography) has been shown to provide efficient possibilities to produce bioactive compounds-enriched fractions [48]. The CPC chromatographic method was used previously for the purification of xanthohumol. In their studies, Renault et al. [49] fractionated the ethanol extract of hop cones (11 g) using quaternary biphasic system composed of heptane/toluene/acetone/water 24.8:2.8:50:22.4 *v/v*. Similarly to Renault et al. [49], in our study, the post-residues hop cones extract E2 (see Figure 2) was fractionated with a quaternary biphasic solvent system. However, ethyl acetate and methanol were used instead of toluene and acetone. For the xanthohumol purification, heptane, ethyl acetate, methanol, and water were mixed in a ratio of 6:5:6:5 *v/v*, respectively. The CPC resulted in the production of xanthohumol-enriched fraction with the xanthohumol concentration of 81.70 wt%, based on HPLC analysis.

### 2.3. Biological Properties

#### 2.3.1. Determination of Growth Inhibition Zones

The crude extract (E1) and xanthohumol (XN) were preliminary tested for their antibacterial activity using modified disc diffusion method. The zones of bacterial growth inhibition were summarized in Table 3. In general, the greater the inhibition zone, the higher sensitivity of bacteria towards analyzed therapeutic agent. The obtained data indicated that both hop samples had activity against Gram-positive bacterial strains, whereas they did not exhibit activity against Gram-negative ones. The tested *P. acne* strains, which are the main reason of seborrheic skin diseases and acne [50], were inhibited by crude extract (E1) and XN in the ranges of 29–26 mm and 15–13 mm, respectively. In turn, zones of growth inhibition measured for caries strains (*S. mutans*, *S. sanguinis*) were 29–28 mm (around extract (E1)) and 21–13 mm (around XN), which also indicated favorable anti-caries activity of the tested hop samples. Thus, it was demonstrated that crude extract (E1) inhibited the growth of all tested Gram-positive strains more potently than xanthohumol. Nevertheless, the antibacterial activity of hop-derived compounds was lower compared to commercial antibiotics.

#### 2.3.2. Determination of MIC and MBC/MIC Ratio

In the next step, the compounds from Marynka hop variety were subjected to evaluation of MIC (minimum inhibitory concentration) and MBC (minimum bactericidal concentration). The MIC values (Table 4) indicated that the inhibition activity of both hop samples against aerobic Gram-positive strains and caries strains (*S. mutans*, *S. sanguinis*) was similar to that of classical antibiotics. Moreover, it was proven that crude extract (E1) possessed greater antibacterial activity against Gram-positive strains that xanthohumol (XN). Thus, extract E1 showed two-fold lower MIC values towards *S. sanguinis PCM 2335*, *P. acnes PCM 2400*, and *P. acnes PCM 2334* compared to XN. In turn, due to the inability to inhibit the growth of Gram-negative strains, the MIC values for extract E1 and XN were not determined.

**Figure 1:**
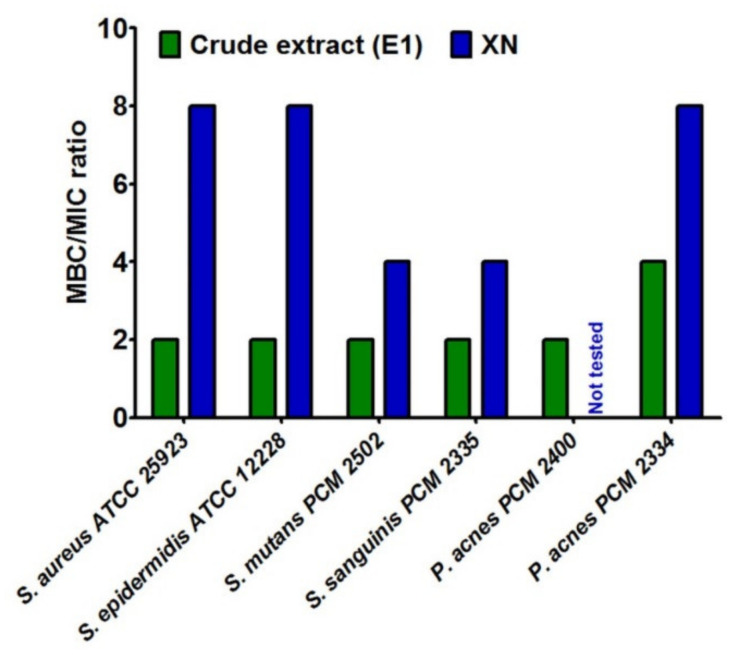
The ratio between minimal bactericidal concentration (MBC) and minimum inhibitory concentration (MIC) for compounds from Marynka hop variety.

Then, the Minimum Bactericidal Concentration (MBC) was determined. It is defined as the lowest concentration of the extract, which has ability to kill 99.9% of organisms. This test also indicated stronger antibacterial nature of crude extract in comparison with XN (Supplementary Table S1). Additionally, to clearly distinguish the inhibiting and killing abilities of tested hop compounds, the ratio between MBC and MIC for selected bacterial strains was determined (Figure 1). It was assumed that MBC/MIC ratio ≤4 denoted bactericidal effect of compounds, while MBC/MIC ratio >4 denoted bacteriostatic one [51]. Thus, crude extract (E1) exhibited bactericidal properties against all tested Gram-positive strains, including acne and caries bacteria. In turn, xanthohumol (XN) mainly possessed bacteriostatic activities. Other authors indicated that, MBC values of phytocompounds from hop exhibited concentration-dependent bactericidal effects [14,18].

It is worth underlining that, unlike Gram-negative bacteria strains, the Gram-positive ones are known to be highly sensitive to hop compounds [7,14,15,18,27,28,29,33], which confirms the results obtained in this research. However, most of studies performed by other scientists indicated better antibacterial effect of xanthohumol compared to another hop components such as humulones or lupulones [7,14,18,33]. In turn, our results clearly proved superior effect of crude extract (E1) in comparison with xanthohumol (XN). Presumably, the reason of this phenomenon is associated with the composition and synergistic effect of active substances in the investigated crude extract (E1). Indeed, it was composed not only of α- and β-acids, but also of terpene derivatives, especially myrcene, β-caryophyllene, β-farnesene, and humulene (Table 1 and Table 2). Thus, it seems that such mixture of hop-derived compounds are more beneficial than xanthohumol alone in terms of bacterial growth inhibition. Other research groups also noted higher activity of extracts of natural origin in comparison with the activity of individual components [52,53], or some authors indicated that a single compound did not have antimicrobial activity, which was visible only in the case of hybrids of active compounds of plant [54].

#### 2.3.3. Determination of Synergistic Effect with Antibiotics

The theoretical synergism between hop compounds and selected antibiotics were determined and the results were summarized in Table 5. It was demonstrated that none of tested samples had an antagonistic effect when it was combined with the selected antibiotic. Importantly, crude extract (E1) showed beneficial synergistic effect (synergistic or at least additive effect) in combination with cefepime, ceftriaxone, ciprofloxacin, and sparfloxacin against all tested Gram-positive strains (FICI ranged for 0.375–0.562). Xanthohumol exhibited synergistic activity with cefepime and ceftriaxone against caries strains—*S. mutans PCM 2502*, *S. sanguinis PCM 2335*, and with cefepime against *P. acnes PCM 2334*. Additivity was noted in the presence of XN with ciprofloxacin for caries strains. Another combinations between hop compounds and antibiotics had no effect against tested Gram-positive strains.

The synergistic effect between hop compounds and some antibiotics has been studied previously by Natarajan et al. [12]. The potential mechanism of synergistic action between these agents resulted from enhanced permeability of cell membrane for antibiotics, causing by the hop compounds. The authors demonstrated positive co-action of β-acids as well as xanthohumol in combination with polymyxin, tobramycin, and ciprofloxacin. Interestingly, xanthohumol exhibited higher synergism with these antibiotics compared to β-acids. In our study, crude extract (E1) possessed better ability to enhace antibacterial activity of antibiotics in comparison with xanthohumol. As mentioned previously, crude extract (E1) exhibited beneficial lower MIC values than xanthohumol, most likely due to its unique composition. Thus, the mixture of hop components contained in the crude extract (E1) also significantly enhanced antibacterial effect of antibiotics.

#### 2.3.4. Determination of Cytotoxicity

After 24-h incubation, it was found that xanthohumol (XN) exhibited a higher cytotoxic effect against normal human fibroblasts (BJ cells) compared to crude extract (E1). Thus, the values of CC_50_ for extract E1 and XN were 155.70 ± 4.23 μg/mL and 26.56 ± 2.62 μg/mL, respectively (Table 6). Taking into account calculated TI values, which determined the potential safety of compounds, the best results were obtained for crude extract (E1). The TI values for XN were approximately 6-fold lower (Table 6). These results indicated that crude extract (E1) possessed beneficial antibacterial activity with slight cytotoxic property. It was considered that compounds which possessed a TI value higher than 10, can be allocated for in vivo study due to suitable antibacterial and cytotoxic activities in vitro [51].

#### 2.3.5. Determination of Anti-Proliferative Activity

The ability of crude extract (E1) and xanthohumol (XN) to inhibit proliferation of some cancer cells and normal ones (control) was also evaluated (Table 7). It was demonstrated that extract E1 exhibited the highest inhibition activity against human hepatocellular carcinoma cells (HepG2), while XN had the greatest anti-proliferative effect towards human lung adenocarcinoma cells (A549). Importantly, normal cells (BJ cell line) incubated with crude extract maintained higher ability to proliferate compared to these cells treated with xanthohumol. The calculated values of selectivity indexes (SI) allowed to determine anti-proliferative potential of tested samples (Table 7). The SI value higher than 1 denotes that tested compound exhibited better selectivity towards cancer cells than normal ones. It was found that crude extract (E1) had SI values > 1 towards all tested cancer cell lines. In turn, XN possessed SI values > 1 for A549 and MCF-7 cell lines.

To date, the anti-proliferative and anticancer activities of compounds from hop have been well-documented in literature [4,8,9,10,11,29,33,55,56,57]. In this case, the particular scientific attention is focused on xanthohumol (XN) and its derivatives. In our study, we demonstrated very promising anti-proliferative activity of both hop samples. Nevertheless, based on obtained SI values, it seems that the mixture of hop-derived compounds (crude extract (E1)) exhibited slightly (MCF-7 cells vs. BJ cells) and significantly (HepG2 cells vs. BJ cells) better anti-proliferative selectivity compared to xanthohumol alone.

## 3. Materials and Methods

### 3.1. Materials

An authentic analytical standard of xanthohumol (98% purity) and TFA (trifluoroacetic acid, ≥99.0%) for UHPLC analysis as well as gallic acid as well as Folin-Ciocalteau reagent for TPC (total phenolic content) were obtained from Sigma-Aldrich Chemicals (Poland). The HPLC grade ethanol (Baker) as well as anhydrous sodium carbonate (Chemsolve) were purchased from Witko (Łódź, Poland). The solvents for CPC purification (ethyl acetate, n-heptane, methanol with p.a grade as well as acetonitrile with LC-MS purity) were purchased from Avantor Performance Materials (Gliwice, Poland). The ICE-3 standard (hop extract containing a specified concentration of α- and β-acids) was purchased from LaborVeritas (Germany). High purity water used in HPLC tests and for the preparation of a two-phase system was obtained on an Aquinity membrapure apparatus (Germany). Carbon dioxide (99.9%, *v/v*), which was used as the mobile phase in SFE, was stored in a CO_2_ installation tank.

In the case of microbial analysis, the following reagents were used: Mueller-Hinton agar or broth (Biomaxima, Poland), Brain-Heart Infusion agar or broth (Biomaxima, Poland), Gentamicin sulfate (Sigma-Aldrich Chemicals, Poland), Ceftriaxone (Polpharma, Poland), Cefepime (Bristol-Myers Squibb, USA), Sparfloxacin (Dainippon Pharmaceutical CO., LTD, Japan), and Ciprofloxacin (Polpharma S.A. Poland). The cell culture experiments were performed using reagents purchased from Sigma-Aldrich Chemicals, Poland: dimethyl sulfoxide (DMSO), human recombinant insulin, penicillin–streptomycin solution, phosphate buffered saline (PBS), sodium dodecyl sulfate (SDS), thiazolyl blue tetrazolium bromide (MTT), and trypsin-EDTA solution (0.25%). Moreover, Eagle’s Minimum Essential Medium (EMEM) and F-12K Medium were obtained from ATCC (UK), while fetal bovine serum (FBS) was supplied by Pan-Biotech (Germany).

### 3.2. Hop Raw Material

The dried hop cones of the “Marynka” variety were purchased from the Polish company Import-Export J. A. Szałas (Karczmiska, Poland). Marynka is a variety of hop with combined aromatic and bitter characteristics, derived from the English variety Brewers Gold. Marynka has a hoppy aroma, quite intense but pleasant [21,58,59]. The moisture content of hop cones was 10.6%.

### 3.3. Supercritical Fluid Extraction (SFE)

**Figure 2:**
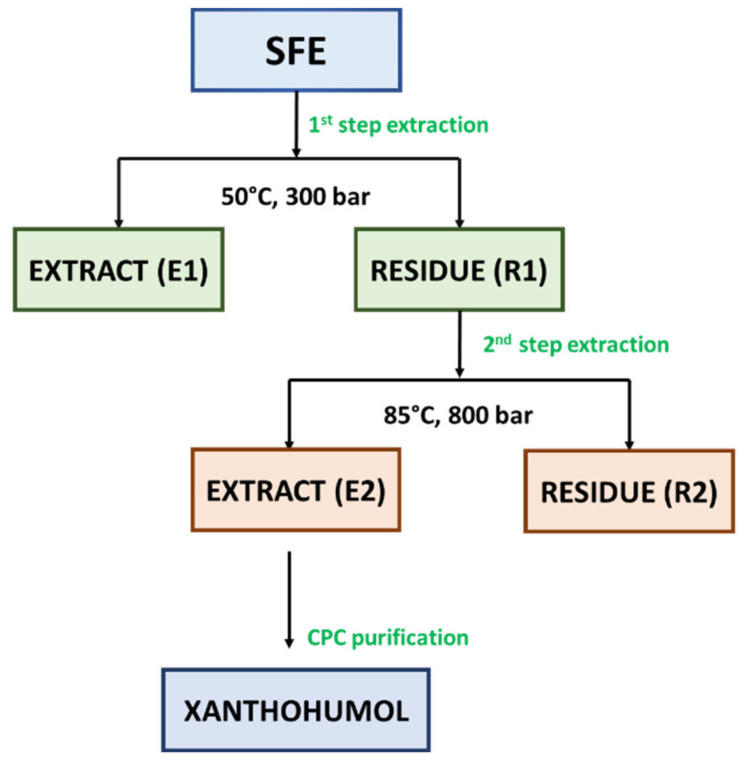
The scheme of the separation and purification of bioactive compounds from Marynka hop cones scCO_2_ extract. Abbreviations: E—extract, CPC—centrifugal partition chromatography, R—residue, SFE—Supercritical fluid extraction.

For the extraction process, hop cones were ground to obtain an average grain size of 0.5 mm, and subjected to palletization. The produced pellets had dimensions 5 × (7–8) mm. The dynamic supercritical fluid extraction (SFE) process was performed on a ¼-technical (the extractor capacity of 40 dm^3^) scale research plant at the Łukasiewicz Research Network—New Chemical Syntheses Institute (Puławy, Poland) operating at temperatures up to 90 °C and pressures up to 1000 bar. Dried and pelleted hop cones (4 kg) were extracted with pure carbon dioxide according to 2-step procedure. As a 1st step, the extraction of hops was carried out at the temperature of 50 °C under the pressure of 250 bar with the consumption of CO_2_ amounting to 50 kg CO_2_/kg of the extraction input. As a result, the crude hop extract (E1) was obtained and the residues (R1), which were subjected to further extraction. As a second step, the post-extraction residues (R1) were re-extracted in order to obtain a xanthohumol rich extract (E2). The following parameters were used: pressure 800 bar, temperature 85 °C, and CO_2_/EtOH (99:1; *v/w*) consumption of 105.1 kg/kg dry weight. The extract E2 was finally purified with the use of preparative chromatography. The extraction and purification of Marynka scCO_2_ extract scheme is presented in Figure 2. Both steps were performed once.

### 3.4. Centrifugal Partition Chromatography (CPC)

CPC was used to purify the E2 extract prior to xanthohumol determination on HPLC. The extract E2, which was obtained during 2nd step extraction was preliminary cleaned with an 80% aqueous methanol solution in the ratio of 1 g of sample per 100 mL of solvent. The resultant suspension was centrifuged, followed by the evaporation of the filtrate to dryness on a rotary evaporator at a water bath temperature of 45 °C and a pressure of 32 mbar. Such prepared extract was further purified in order to obtain fraction enriched in xanthohumol. For this purpose, centrifugal partition chromatography (CPC) was applied with the use of ARMEN SPOT PREP chromatograph (Armen, France) equipped with two rotors (with the capacity of 250 and 1000 mL) and UV-Vis detector.

The separation was carried out using the P system from Arizona solvent system (commonly used solvents combinations in CPC) on a 250 mL rotor. Heptane, ethyl acetate, methanol, and water were mixed in a ratio of 6:5:6:5 *v/v*, respectively. From the prepared solvent system, 5 mL of the each upper and lower phases were pipetted into a jar with a ready extract (50 mg), which was filtered and placed in the injection valve. The ascending mode (ASC), in which the lighter (upper) phase was the mobile one was applied. After starting the apparatus and the software with the developed method, the entire system was rinsed with the solvents included in the system. The 10 mL sample dosing loop onto the system was rinsed with methanol. The column was filled with the stationary phase (lower) at the solvent flow rate 30 mL/min and a column speed of 500 rpm. Next equilibration of the column with mobile phase was performed at a solvent flow rate of 8 mL/min and a column speed of 2000 rpm. After the equilibrium was achieved, the injection of the sample was introduced. The elution was performed with a mobile phase flow rate of 8 mL/min and a column speed of 2000 rpm. The column effluent was monitored by a UV-Vis detector with a detection wavelength of 370 nm. The fractions (approx. 14 mL) of CPC run were collected in 20 mL test tubes followed by the evaporation of solvents from fractions collected in 30–40 min of the CPC separation (fractions number 17–23) and HPLC analysis of xanthohumol.

### 3.5. Gas Chromatography Equipped with Mass Spectrometer (GC-MS)

The qualitative analysis of crude extract (E1) in terms of terpenes and volatile compounds was performed with a GC-MS instrument (Agilent 7890) equipped with mass spectrometry and NIST 2011 MS spectra library. For the acquired results, Masshunter software (ver. C.01.03) was used. The following analyses parameters were used for Humulus lupulus Polish “Marynka” extract sample: Agilent Capillary GC column DB-EUPAH (60 m × 250 µm; 0.25 µm; Agilent J&W, Santa Clara, United States) with helium as the carrier gas, and flow rate of 2 mL/min in a split injection mode (40:1). Oven temperature was set initially at 50 °C (held for 1 min) and increased to 260 °C at the rate of 6 °C/min (held for 6 min) and then finally reached 300 °C at the rate of 3 °C/min and held for 20 min. The system was operating in the EI mode (electron energy 70 eV) with the temperature of the source ion set to 230 °C and the scanning range from 40 to 650 amu. The analysis time was 75 min. The identification of the bioactive compounds was performed based on the comparison of analytes mass spectrum with NIST spectra library. The sample E1 was analyzed in triplicates.

### 3.6. Ultra High Performance Liquid Chromatography (U-HPLC)

The quantitative analysis of α- and β-acids in crude extract (E1) was conducted on Thermo Scientific ACELLA 1200 Ultra High Liquid Chromatography system (Thermo Scientific, Waltham, MA, USA) equipped with UV-Vis detector. Chromatographic separation of the α- and β-acids was carried out at the temperature 35 °C on Thermo Scientific Hypersil Gold C18 column with the dimensions of 200 × 2.1 mm; 1.9 µm. Both water (solvent A) and acetonitrile (solvent B) with the addition of 0.1% TFA (*v/v*) to each phase were used as the components of the mobile phase in a gradient mode. The composition of the mobile phase was 0–14 min 40% A, 14–18 min 16% A, 18–28 min 40% A, 28–30 min 40% A with the column temperature of 35 °C, mobile phase flow rate of 400 µL/min, sample injection volume of 2 µL and the detection at 314 nm.

For the determination of xanthohumol in extract E2 and CPC fractions, the same system was used with Hypersil Gold C18 column (200 × 2.1 mm; 1.9 µm) with the temperature of 40 °C, sample injection volume of 2 μL, mobile phase flow rate of 400 µL/min and the wavelength of 370 nm. The composition of the mobile phase was acetonitrile +0.1% TFA (solvent A), water +0.1% TFA (solvent B), gradient elution: 0–13 min 51% A, 13–15 min 95% A, 15–20 min 51% A. Each sample was analyzed in triplicates.

### 3.7. Total Phenolic Content

Total phenolic content in E1 and E2 was determined spectrophotometrically using Folin-Ciocalteau reagent according to the method described in the literature [60,61]. The ethanolic extract solution (50 μL; approximately 10 mg/mL) filtered through a 0.45 μm filter was mixed with distilled water (1.58 mL) and Folin-Ciocalteu reagent (100 μL). After 30 min. an aqueous solution of sodium carbonate (300 μL) was added. After 1 h at 25 °C in the dark absorbance was measured at 765 nm using a UV-Vis spectrophotometer (Jasco V-650, Germany). As for the standard, gallic acid was used and the results were provided as milligram gallic acid equivalent (mgGAE/g extract). The stock solution of gallic acid (10 mg) was prepared in ethanol (2 mL). A calibration curve was determined with concentrations of 0.1, 0.2, 0.3, 0.4, and 0.5 mg/mL.

### 3.8. Antibacterial Activity

#### 3.8.1. Bacterial Strains

The crude extract (E1) and xanthohumol (XN) were subjected for evaluation of their in vitro antibacterial activity. For this purpose the following bacteria were used: aerobic Gram-positive strains (*Staphylococcus aureus ATCC 25923*, *Staphylococcus epidermidis ATCC 12228*), aerobic Gram-negative strains (*Escherichia coli ATCC 25992*, *Pseudomonas aeruginosa ATCC 27853*) supplied by ATCC, United Kingdom and microaerobic Gram-positive strains (*Propionibacterium acnes PCM 2400*, *Propionibacterium acnes PCM 2334*, *Streptococcus mutans PCM 2502*, *Streptococcus sanguinis PCM 2335* purchased from Polish Collection of Microorganisms PCM Institute of Immunology and Experimental Therapy Polish Academy of Sciences, Wroclaw, Poland). The Mueller-Hinton agar or broth (MH-agar, MH-broth) for aerobic strains and Brain-Heart Infusion agar or broth (BHI-agar, BHI-broth) for microaerobic strains were used. Bacterial inoculums were prepared by culturing microorganisms into MH-agar or BHI-agar for 24 or 48 h at 37 °C, respectively. The bacteria were collected and suspended in 0.9% NaCl to obtain density of 0.5 McFarland (1.5 × 10^8^ CFU/mL (CFU: colony forming unit)).

#### 3.8.2. Agar Disc Diffusion Assay

An initial antibacterial character of crude extract (E1) and xanthohumol (XN) was determined by a disc diffusion method [51]. The bacterial inoculum was spread by the cotton swab on the surface of the Petri plates containing required agar. Then, solutions of tested samples (100 µg/mL in DMSO) were placed on inoculated Petri plates. As a control, the solutions of commercially available antibiotics at the same concentrations were used. The plates with MH-agar (for aerobic strains) were incubated for 24 h at 37 °C and the plates with BHI-agar for 48 h at 37 °C. After incubation, the diameter of the growth inhibition zone (in millimetres) around each compound was measured.

#### 3.8.3. MIC and MBC Tests

The minimum inhibitory concentration (MIC) of crude extract (E1) and xanthohumol (XN) was determined only for bacterial strains, which exhibited the bacterial growth inhibition zones (determined during Agar disc diffusion assay, Section 3.8.2). The test was performed using double serial microdilution in the 96-well microtiter plates according to CLSI method with some modifications [62]. The 200 µL of broth was pipetted into each well. The double serial dilutions of tested samples were prepared in the test wells at concentrations ranged 2000 µg/mL–0.098 µg/mL. Finally, 2 µL of tested bacteria inoculum were added to the wells (except for negative sterility control and blanc dye control). The assays were performed for 24 h at 37 °C (aerobic strains) or 48 h at 37 °C (microaerobic strains). After incubation, the panel was digitally analysed at 600 nm. The growth intensity in each well was compared with the negative, positive, and dye controls. Additionally, MBC (minimal bactericidal concentration) was determined. For this purpose, 10 µL of medium from wells without bacterial growth after MIC test were spread on fresh agar. The plates were incubated in suitable conditions and the MBC was defined as the lowest concentration of sample without bacterial growth. Each experiment was repeated in triplicate.

#### 3.8.4. Synergy Test

The synergistic interactions between crude extract (E1), xanthohumol (XN), and antibiotics (gentamicin, ceftriaxone, cefepime, sparfloxacin, and ciprofloxacin) were examined by the checkerboard test, based on MIC determination. The stock solutions and serial twofold dilutions of antibiotics as well as plant samples to fourfold the MIC were prepared. A total of 50 μL of broth was distributed into each well of the microdilution plates. The first tested antibiotic of the combination was serially diluted vertically, while the plant sample was diluted horizontally in the 96-well plate. Next, 100 μL of bacterial inoculum (1.5 × 10^8^ CFU/mL) were added and the plates were incubated in suitable conditions. The resultant checkerboard contained each combination of two studied agents. The plate layout also included the MIC determination of agents used separately. The fractional inhibitory concentration index (FICI) was calculated for each combination of two agents concentrations, according to the following formula:

FICI = (MIC_A/B_/MIC_A_) + (MIC_B/A_/MIC_B_) (1)

where MIC_A_ denotes MIC of the agent A alone, MIC_A/B_ denotes the MIC of agent A in combination with agent B and MIC_B_, and MIC_B/A_ is defined analogously as agent A.

The obtained FICI values indicated the following effects: synergy (FICI ≤ 0.5), additivity (FICI: 0.5–1.0), indifference (FICI: 1.0–4.0), or antagonism (FICI > 4) between two agents [63].

### 3.9. Cell Culture Experiments

#### 3.9.1. Cell Lines

Cytotoxicity evaluation was performed using normal human skin fibroblast (BJ cell line, ATCC^®^ CRL-2522^TM^). In turn, anti-proliferative activity was assessed towards cancer cells: human lung adenocarcinoma (A549, ATCC^®^ CCL-185^TM^), human hepatocellular carcinoma (HepG2 cell line, ATCC^®^ HB-8065^TM^), and human breast adenocarcinoma (MCF-7, ATCC^®^ HTB-22^TM^) as well as towards normal cells (BJ cell line was used as model of normal cells). All cell lines were purchased from ATCC, United Kingdom and cultured according to manufacturer recommendations.

#### 3.9.2. Cytotoxicity

Firstly, 100 μL of BJ cell suspension at concentration of 1.5 × 10^5^ cells per ml was seeded in 96-well plates. The plates were transferred to the incubator for 24 h (37 °C, 5% CO_2_) to allow cell attachment. On the next day, the two-fold serial dilutions of crude extract (E1) and xanthohumol (XN) were prepared in culture medium. The final tested concentrations were 1000–1.95 μg/mL (crude extract (E1)) and 250–0.488 μg/mL (XN). Then, the culture medium was gently discarded and dilutions of samples were added to the cells. After 24-h incubation (37 °C, 5% CO_2_), the BJ cell viability was assessed via MTT test according to our protocol described earlier [51]. Taking into account results obtained with the MTT assay, the values of CC_50_ were calculated using 4-parameter nonlinear regression analyses (GraphPad Prism 5, version 5.04.). The CC_50_ denotes the concentration that caused reduction of BJ cell viability to 50%. Moreover, in order to determine the potential safety of plant samples, TI values were determined. The TI (therapeutic index) was calculated by division of CC_50_ value by MIC value (determined during antibacterial assay, Section 3.8.3).

#### 3.9.3. Anti-Proliferative Properties

At the beginning of experiment, 100 μL of cell suspensions in a complete growth medium at concentration of 4 × 10^4^ cells per ml (A549), 4 × 10^5^ cells per ml (HepG2), 3 × 10^5^ cells per mL (MCF-7), and 4 × 10^4^ cells per ml (BJ) were seeded on 96-well plates. The plates were transferred to the incubator for 24 h (37 °C, 5% CO_2_) to allow cell attachment. On the next day, the two-fold serial dilutions of crude extract (E1) and xanthohumol (XN) were prepared in culture media. The final tested concentrations were 1000–1.95 μg/mL (crude extract E1) and 250–0.488 μg/mL (XN). Then, the culture media were gently discarded and dilutions of extracts were added to the cells. After a 72-h incubation (37 °C, 5% CO_2_), the cell ability to proliferate was assessed via MTT assay [51]. Taking into account results obtained with MTT assay, the values of IC_50_ were calculated using 4-parameter nonlinear regression analyses (GraphPad Prism 5, version 5.04.). The IC_50_ denotes concentration required to restrain cell proliferation in 50%. Moreover, in order to determine the selectivity of extracts, SI values were determined. The SI (selective index) was calculated by the division of IC_50_ value obtained for normal cells (BJ cell line) by IC_50_ values obtained for cancer cells (A549, HepG2, MCF-7 cell lines).

## 4. Conclusions

In this study, two plant samples, namely crude extract (E1) and pure xanthohumol, were obtained from the Polish “Marynka” hop variety using an efficient two-step supercritical fluid extraction method. Our data clearly indicated that crude extract (E1), composed of α- and β-acids as well as terpene derivatives, especially myrcene, β-caryophyllene, β-farnesene, and humulene, had better antibacterial activity and exhibited a higher synergistic effect with commercial antibiotics compared to those of xanthohumol. Moreover, extract E1 was less cytotoxic than XN, and as a consequence possessed higher values of therapeutic indexes, which indicated its better potential safety in vitro. Crude extract (E1) was also found to have more selective anti-proliferative activity towards cancer cells than towards normal ones. Thus, it should be underlined that these specified mixtures of hop compounds contained in crude extract (E1) may have better pro-health action compared to xanthohumol. Therefore, as proposed by us, this highly efficient two-stage extraction method with supercritical fluid for the production of bioactive plant compounds is recommendable. It should be noted that this method fits into an increasing global trend in green extraction, allowing for enhancement of the composition of the bioactive compound. Thus, this study sheds new light on obtaining bioactive compounds from hop, which can be used in pharmacy and medicine.

## Figures and Tables

**Table 1:** The quality profile (% peak area) of crude extract (E1) obtained from Polish “Marynka” hop variety.

Compound	Retention Time, min	Peak Area Percentage, %
citronelol	11.87	0.58 ± 0.04
myrcene	12.33	20.51 ± 0.27
linalool	16.01	0.39 ± 0.03
copaene	22.09	0.21 ± 0.02
α-Bergamotene	23.32	0.35 ± 0.11
β-caryophyllene	23.72	4.88 ± 0.66
β-farnesene	23.77	4.69 ± 0.09
humulene	24.74	18.3 ± 0.38
α-cedrene	24.87	0.45 ± 0.09
β-guajen	24.98	0.23 ± 0.21
valencen	25.58	0.29 ± 0.08
δ-cadinene	26.52	0.98 ± 0.08
humulen epoxy	29.45	0.45 ± 0.05

**Table 2:** The chemical composition of obtained extract (E1 and E2) and CPC fraction.

Parameter	1 Step Extraction (Crude Extract E1)	2 Step Extraction (Extract E2, CO_2_^b^ or CO_2_/EtOH^c^)	CPC Fractionation
α-acids, % *m/m*	42.48 ± 0.68	n.a. ^a^	n.a. ^a^
β-acids, % *m/m*	19.07 ± 0.30	n.a. ^a^	n.a. ^a^
xanthohumol, wt%,	n.a. ^a^	4.80 ± 0.58 ^c^	81.70 ± 0.66
	n.a. ^a^	6.50 ± 0.43 ^b^ [1]	n.a ^a^
TPC, mgGAE/g	n.a. ^a^	52.19 ± 1.11 ^c^	n.a. ^a^
	n.a. ^a^	48.71 ± 0.76 ^b^	n.a. ^a^
extraction yield, wt%	11.40	4.64 ^b^	n.a. ^a^
		10.76 ^c^	n.a. ^a^

^a^ n.a.—not applicable; ^b^ CO_2_—extraction with pure carbon dioxide; ^c^ CO_2_/EtOH—extraction with carbon dioxide modified with EtOH (96%) (99:1, *v/w*).

**Table 3:** Zones of bacterial growth inhibition of compounds from Marynka hop variety and selected antibiotics.

Bacteria	Zones of Bacterial Growth Inhibition [mm]						
	Crude Extract (E1)	XN	Gentamicin	Ceftriaxone	Cefepime	Sparfloxacin	Ciprofloxacin
*S. aureus* *ATCC 25923*	27	13	36	30	37	39	34
*S. epidermidis* *ATCC 12228*	35	17	38	44	46	47	47
*E. coli* *ATCC 25992*	6	0	30	37	38	36	35
*P. aeruginosa* *ATCC 27853*	0	0	41	28	10	38	40
*S. mutans* *PCM 2502*	29	13	36	30	29	30	43
*S. sanguinis* *PCM 2335*	28	21	30	44	42	44	44
*P. acnes* *PCM 2400*	26	13	36	36	37	34	40
*P. acnes* *PCM 2334*	29	15	38	36	39	30	37

**Table 4:** Minimum inhibitory concentration (MIC) of compounds from Marynka hop variety and selected antibiotics.

Bacteria	Minimum Inhibitory Concentration (MIC) [μg/mL]						
	Crude Extract (E1)	XN	Gentamicin	Ceftriaxone	Cefepime	Sparfloxacin	Ciprofloxacin
*S. aureus* *ATCC 25923*	0.195	0.195	0.098	0.195	0.781	0.049	0.049
*S. epidermidis* *ATCC 12228*	0.098	0.098	0.098	0.195	0.195	0.098	0.049
*E. coli* *ATCC 25992*	NT^a^	NT ^a^	1.563	15.63	15.63	15.63	15.63
*P. aeruginosa* *ATCC 27853*	NT^a^	NT ^a^	0.049	12.5	1.563	1.563	1.563
*S. mutans* *PCM 2502*	0.391	0.391	0.098	6.25	0.195	0.049	1.96
*S. sanguinis* *PCM 2335*	0.781	15.625	0.049	6.25	0.195	0.098	0.781
*P. acnes* *PCM 2400*	15.625	62.5	0.049	3.12	1.563	0.098	0.049
*P. acnes* *PCM 2334*	15.625	31.25	0.049	3.12	0.195	1.96	0.781

^a^ NT—not tested due to weak or no activity during agar disc diffusion assay.

**Table 5:** Interactions between compounds from Marynka hop variety and selected antibiotics.

	FICI ^a^ Index of Different Combination of Antibiotics and Hop Compounds									
	Crude Extract (E1)					Xanthohumol (XN)				
Bacteria	gentamicin	cefepime	ceftriaxone	ciprofloxacin	sparfloxacin	gentamicin	cefepime	ceftriaxone	ciprofloxacin	sparfloxacin
*S. aureus* *ATCC 25923*	1.5	0.5	0.562	0.5	0.562	1.06	2	1.06	1.5	1.5
*S. epidermidis* *ATCC 12228*	1.5	0.5	0.562	0.562	0.5	1.5	1.06	1.06	1.5	1.06
*S. mutans* *PCM 2502*	1.5	0.562	0.375	0.5	0.375	1.06	0.5	0.5	0.562	1.06
*S. sanguinis* *PCM 2335*	1.06	0.5	0.375	0.375	0.562	1.5	0.5	0.375	0.562	1.06
*P. acnes* *PCM 2400*	2	0.562	0.562	0.562	0.562	1.5	0.562	0.562	1.06	1.06
*P. acnes* *PCM 2334*	1.5	0.5	0.375	0.5	0.5	1.5	0.5	0.562	1.06	1.06

^a^ Fractional inhibitory concentration index. FICI Index range: ≤0.5—synergy; 0.5 to 1.0—additivity; 1.0 to 4.0—indifference; >4.0—antagonism.

**Table 6:** Cytotoxicity and therapeutic index of compounds from Marynka hop variety.

Bacteria	Crude Extract (E1) CC_50_^a^ = 155.70 ± 4.23 μg/mL	Xanthohumol (XN) CC_50_^a^ = 26.56 ± 2.62 μg/mL
	TI ^b^ (CC_50_/MIC)	
*S. aureus* *ATCC 25923*	789	136
*S. epidermidis* *ATCC 12228*	1589	271
*S. mutans* *PCM 2502*	398	67.90
*S. sanguinis* *PCM 2335*	199	1.69
*P. acnes* *PCM 2400*	9.96	0.42
*P. acnes* *PCM 2334*	9.96	0.84

^a^ CC_50_—cytotoxic concentration that caused reduction of fibroblast viability to 50%. ^b^ TI- therapeutic index denotes potential safety of plant samples. It is calculated as a ratio between CC_50_ and MIC (based on data from Table 4).

**Table 7:** The anti-proliferative activity and selectivity index of compounds from Marynka hop variety.

Cell Line	Crude Extract (E1)		Xanthohumol (XN)	
	IC_50_^a^ [μg/mL]	SI ^b^	IC_50_^a^ [μg/mL]	SI ^b^
A549	45.17 ± 3.58	2.30	7.39 ± 2.99	2.33
HepG2	26.27 ± 1.56	3.97	36.36 ± 3.48	0.47
MCF-7	66.48 ± 2.97	1.56	12.18 ± 2.89	1.42
BJ	104.30 ± 4.16	-	17.25 ± 1.35	-

^a^ IC_50_—concentration of compound required to inhibit cell proliferation to 50%. ^b^ SI- selective index. The value was calculated as a ratio between IC_50_ for normal cells (BJ) and IC_50_ for cancer cells (A549, HepG2 or MCF-7).

## Data Availability

Data available on request.

## Supplementary Materials

The following are available online, Table S1: Minimum bactericidal concentration (MBC) of compounds from Marynka hop variety.

## Sample Availability

Samples of the compounds—crude extract (E1) and xanthohumol are available from the authors (Katarzyna Tyśkiewicz, Agnieszka Dębczak, Edward Rój).
