# Supplementary material for: Bioactive Compounds Obtained from Polish “Marynka” Hop Variety Using Efficient Two-Step Supercritical Fluid Extraction and Comparison of Their Antibacterial, Cytotoxic, and Anti-Proliferative Activities In Vitro
Source: Molecules. 2021 Apr 19;26(8):2366. doi: 10.3390/molecules26082366 (PMC8073632; doi:10.3390/molecules26082366)
Supplement: Supplementary file 1 [file molecules-26-02366-s001.zip › molecules-1170699-supplementary.pdf]

SUPPLEMENTARY

Supplementary Table S1. Minimum bactericidal concentration (MBC) of compounds from Marynka hop variety

**Bacteria**	**Minimum bactericidal concentration (MBC) [µg/ml]**	
	Crude extract (E1)	XN
S. aureus ATCC 25923	0.391	1.564
S. epidermidis ATCC 12228	1.96	1.564
S. mutans PCM 2502	0.781	1.564
S. sanguinis PCM 2335	1.562	62.5
P. acnes PCM 2400	31.25	ND[a]
P. acnes PCM 2334	62.5	250

[a] ND– not detected in tested concentration range
